# Alkhurma Hemorrhagic Fever Virus RNA in *Hyalomma rufipes* Ticks Infesting Migratory Birds, Europe and Asia Minor

**DOI:** 10.3201/eid2405.171369

**Published:** 2018-05

**Authors:** Tove Hoffman, Mats Lindeborg, Christos Barboutis, Kiraz Erciyas-Yavuz, Magnus Evander, Thord Fransson, Jordi Figuerola, Thomas G.T. Jaenson, Yosef Kiat, Per-Eric Lindgren, Åke Lundkvist, Nahla Mohamed, Sara Moutailler, Fredrik Nyström, Björn Olsen, Erik Salaneck

**Affiliations:** Uppsala University, Uppsala, Sweden (T. Hoffman, M. Lindeborg, T.G.T. Jaenson, Å. Lundkvist, B. Olsen, E. Salaneck);; Hellenic Ornithological Society/Birdlife, Athens, Greece (C. Barboutis);; Ondokuz Mayis University, Samsun, Turkey (K. Erciyas-Yavuz);; Umeå University, Umeå, Sweden (M. Evander, N. Mohamed);; Swedish Museum of Natural History, Stockholm, Sweden (T. Fransson);; Estación Biológica de Doñana, Sevilla, Spain (J. Figuerola);; Ciber Epidemilogía y Salud Pública, Madrid, Spain (J. Figuerola);; Hebrew University of Jerusalem, Jerusalem, Israel (Y. Kiat);; Linköping University, Linköping, Sweden (P.-E. Lindgren, F. Nyström);; Agence Nationale de Sécurité Sanitaire de l’Alimentation, Maisons-Alfort, France (S. Moutailler)

**Keywords:** Europe, Greece, west Asia, Asia Minor, Turkey, vector-borne infections, viral hemorrhagic fevers, Alkhurma hemorrhagic fever, viruses, flavivirus, Alkhurma hemorrhagic fever virus, Kyasanur Forest disease virus, ticks, *Hyalomma marginatum* sensu lato, *Hyalomma rufipes*, birds, passerine birds, sedge warbler, eastern woodchat shrike, western yellow wagtail, common redstart, *Acrocephalus schoenobaenus*, *Lanius senator niloticus*, *Motacilla flava*, *Phoenicurus phoenicurus*

## Abstract

Alkhurma hemorrhagic fever virus RNA was detected in immature *Hyalomma rufipes* ticks infesting northward migratory birds caught in the North Mediterranean Basin. This finding suggests a role for birds in the ecology of the Alkhurma hemorrhagic fever virus and a potential mechanism for dissemination to novel regions. Increased surveillance is warranted.

Alkhurma hemorrhagic fever virus (AHFV) was identified in 1995 after an outbreak of viral hemorrhagic fever in Jeddah Province, Saudi Arabia (*1*). This virus is a variant of Kyasanur Forest disease virus (KFDV), which is endemic in eastern India, and a member of the mammalian tickborne flaviviruses (*2*). An association between Alkhurma hemorrhagic fever cases and livestock handling was identified early on, suggesting goats and sheep are potential reservoirs (*3*). Furthermore, AHFV has been identified in *Ornithodoros savignyi* soft ticks and *Hyalomma dromedarii* hard ticks (potential vectors) in Saudi Arabia, both of which are associated with camels (potential reservoir) (*4*,*5*). Alkhurma hemorrhagic fever is endemic in several provinces of Saudi Arabia; sporadic cases have been reported in Africa near the Egypt–Sudan border and cases of seropositivity in Djibouti (*6**–**8*) (Figure 1). AHFV RNA has also been detected in *Amblyomma lepidum* ticks collected from cattle in Djibouti (*9*).

**Figure 1:**
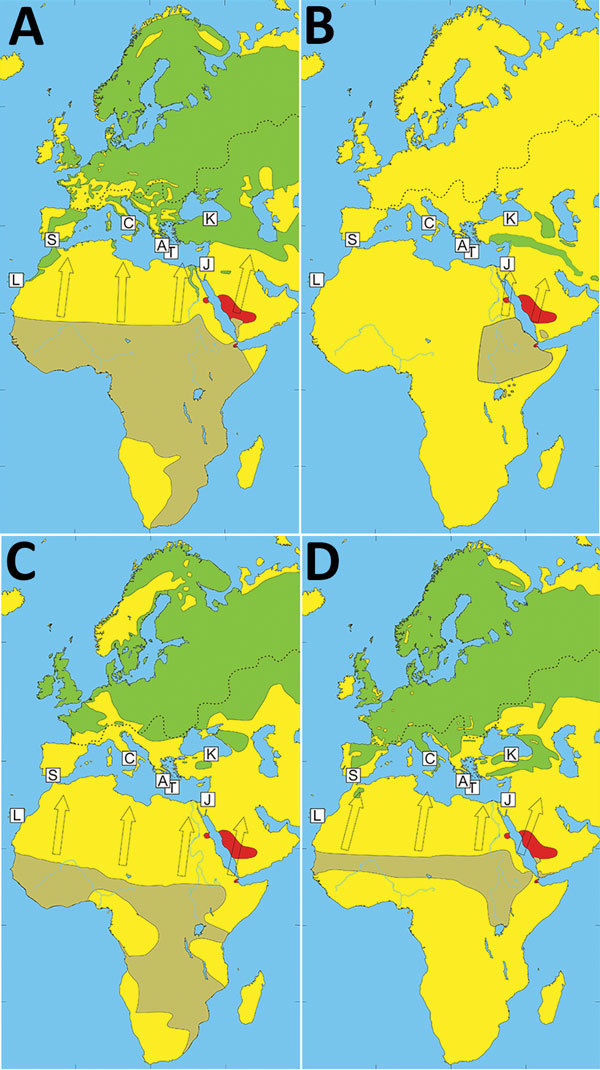
Wintering (light brown) and breeding (green) locations and springtime migratory routes (arrows) of birds testing positive for Alkhurma hemorrhagic fever virus (AHFV) RNA in Greece and Turkey, 2010 and 2014. The 4 bird species found infested by *Hyalomma* ticks carrying AHFV RNA were the western yellow wagtail (*Motacilla flava*) (A), eastern woodchat shrike (*Lanius senator niloticus*) (B), sedge warbler (*Acrocephalus schoenobaenus*) (C), and common redstart (*Phoenicurus phoenicurus*) (D). Red shading indicates areas where AHFV has been detected. The dashed line shows the approximate northern geographic boundary of *H. marginatum* complex ticks (based on information from the European Center for Disease Prevention and Control, https://ecdc.europa.eu/en/disease-vectors/surveillance-and-disease-data/tick-maps). *H. rufipes* ticks have a wide geographic distribution in Africa and are present in Saudi Arabia. Collection sites are labeled: Andikíthira, Greece (A); Capri, Italy (C); Jerusalem, Israel (J); Kizilirmak Delta, Turkey (K); Huelva and Sevilla Provinces, Spain (S); Canary Islands, Spain (L); and Crete, Greece (T). Maps created based on information from The Birds of the Western Palearctic, volumes 5–7, Oxford (UK): Oxford University Press; 1988, 1992, 1993.

The clinical manifestation of Alkhurma hemorrhagic fever resembles that of other viral hemorrhagic fevers: initial malaise and influenza-like symptoms, followed by encephalitis, icterus, and ecchymosis. Case fatality is ≈25% but could be considerably lower, considering mild cases are probably undiagnosed (*1*). It has been suggested that both KFDV and AHFV originated in Africa and that, subsequently, KFDV spread to India and AHFV to Saudi Arabia, KFDV possibly disseminating farther to southern China by migratory birds (*2*). Also, other pathogens have been found to disseminate by means of ticks on migratory birds (*10*). In light of these findings, case reports in Africa (*6**–**8*), and increasing case frequency in Saudi Arabia (*11*), we investigated whether ticks infesting migratory birds en route from Africa to Europe and Asia during springtime carry AHFV.

## The Study

**Figure 2:**
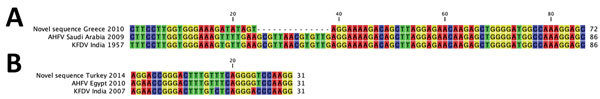
Nucleotide alignments of novel AHFV sequences obtained from *Hyalomma marginatum* sensu lato ticks (likely *H. rufipes*) with reference AHFV and KFDV sequences. A) Partial alignment of 5′ untranslated region of AHFV obtained from tick collected from bird in Andikíthira, Greece, 2010, with corresponding reference sequences of AHFV (GenBank accession no. JF416957) and KFDV (GenBank accession no. HM055369). B) Partial alignment of premembrane sequence of AHFV obtained from tick collected from bird in Turkey, 2014, with corresponding reference sequences of AHFV (GenBank accession no. JX914663) and KFDV (GenBank accession no. JQ434075). AHFV, Alkhurma hemorrhagic fever virus; KFDV, Kyasanur Forest disease virus.

We collected ticks from birds migrating northward initially at 2 bird observatories on the Mediterranean islands Capri (Italy; 40°33′N, 14°15′E) and Andikíthira (Greece; 35°51′N, 23°18′E) during spring of 2009 and 2010. We captured 14,824 birds (78 species) during their yearly migration, presumably leaving Africa for breeding grounds in Europe or Asia. We collected 747 ticks, 88% of which were identified morphologically as members of the complex *H. marginatum* sensu lato (s.l.), most likely *H. rufipes* and *H. marginatum*. We screened cDNA for AHFV by real-time PCR with primers targeting the 5′ untranslated region of AHFV (*12*). Five fully engorged ticks, morphologically and molecularly determined to be *H. marginatum* s.l., likely *H. rufipes* (GenBank accession nos. MH061004–MH061008, MH061010–MH061014), tested positive for AHFV RNA in 2 separate analyses. These 5 ticks (4 nymphs, 1 larva) were collected at Andikíthira in 2010 (Table) from 3 bird species that winter in sub-Saharan Africa and breed in Europe (Figure 1, panels A–C). One sedge warbler (*Acrocephalus schoenobaenus*) carried 2 AHFV-positive nymphs. Sequencing of the 5′ untranslated region amplicons revealed 2 identical 72-bp sequences. BLAST (http://www.ncbi.nlm.nih.gov/blast/) and comparative analyses revealed high identity to AHFV and KFDV reference sequences, despite a region with 7 consecutive mismatching nucleotides and a 14-nt deletion (Figure 2, panel A). Apart from this difference, 100% identity was seen with all other AHFV and KFDV sequences available in GenBank.

**Table:** Characteristics of birds infested by ticks testing positive for Alkhurma hemorrhagic fever virus RNA by real-time PCR, Greece and Turkey, 2010 and 2014

Ring no.	Bird species	Capture date	Tick species	Life stage	Capture site
B913855	Eastern woodchat shrike (*Lanius senator niloticus*)	2010 May 5	*Hyalomma marginatum* sensu lato (*H. rufipes*)	Nymph	Greece
A228919	Sedge warbler (*Acrocephalus schoenobaenus*)	2010 May 7	*H. marginatum* s.l. (*H. rufipes*)	Nymph	Greece
A225683	Western yellow wagtail (*Motacilla flava*)	2010 May 9	*H. marginatum* s.l. (*H. rufipes*)	Larva	Greece
A225166	Sedge warbler (*A. schoenobaenus*)	2010 May 13	*H. marginatum* s.l. (*H. rufipes*)	Nymph	Greece
A225166	Sedge warbler (*A. schoenobaenus*)	2010 May 13	*H. marginatum* s.l. (*H. rufipes*)	Nymph	Greece
JB53791	Common redstart (*Phoenicurus phoenicurus*)	2014 Apr 29	*H. marginatum* s.l. (*H. rufipes*)	Adult	Turkey

Because of these results, we included additional bird observatories and collection sites along with Capri and Andikíthira in spring of 2014 and 2015: the Anapodaris River, Crete, Greece (34°59′N, 25°17′E); Huelva Province, Spain (37°30′N, 5°30′W); Sevilla Province, Spain (37°33′N, 6°55′W); Canary Islands, Spain (28°9′N, 15°25′W); Jerusalem, Israel (31°47′N, 35°13′E); and the Kizilirmak Delta, Turkey (41°38′N, 36°05′E). We ringed 22,069 birds (137 species) and collected 1,024 ticks. RNA was extracted at Uppsala University (Uppsala, Sweden) and sent to Agence Nationale de Sécurité Sanitaire de l’Alimentation (Paris, France) for high-throughput screening by microfluidic real-time PCR (Biomark Dynamic Arrays, Fluidigm, South San Francisco, CA, USA) targeting multiple tickborne viruses (S. Moutailler, unpub. data). One fully engorged adult tick, molecularly determined to be *H. marginatum* s.l. (GenBank accession nos. MH061009, MH061015), likely *H. rufipes*, collected from a common redstart (*Phoenicurus phoenicurus*) in Turkey tested positive for AHFV RNA (Table) when using a set of primers and probe that amplifies part of the KFDV/AHFV premembrane region (Kyasanur_poly_F, 5′-ACACGATGCACACACCTGC-3′; Kyasanur_poly_R, 5′- CACCAATGAAACTCTAGTCGTC-3′; Kyasanur_poly_P, 5′-AGAACCGGGACTTTGTCTCAGGGAC-3′). To confirm this finding, a subsequent real-time PCR was performed with the same primers and probe. Alignment of the 31-bp fragment revealed a single nucleotide change compared with available AHFV sequences and 3 or 4 differences compared with available KFDV sequences (Figure 2, panel B). The methods used did not enable virus isolation and propagation; therefore, acquisition of further sequence information proved difficult. The common redstart also breeds in Europe and winters in sub-Saharan Africa, similarly to the 3 previously mentioned bird species (Figure 1, panel D).

## Conclusions

We detected AHFV/KFDV RNA in 6 of 1,771 investigated ticks. Although we could not differentiate between KFDV and AHFV on the basis of the short sequences analyzed, we suggest these sequences represent AHFV because this supposition is geographically and ecologically more plausible. Our findings are insufficient to establish the role of the *H. rufipes* tick as an AHFV vector. However, detection of AHFV RNA in Europe and Asia Minor raises concerns for conceivable dissemination of the virus to these areas, facilitated by climate change resulting in increased distribution of *Hyalomma* spp. ticks.

*H. rufipes* ticks are widely distributed in Africa and are also present on the Arabian Peninsula, along the Red Sea coast (*13*). The *H. rufipes* species is a 2-host tick; that is, the nymph remains attached to ingest blood on the same host it did for its first blood meal as a larva. Immature ticks usually infest birds or small mammals (e.g., hares), whereas adult ticks feed on larger mammals (e.g., camels, buffaloes, cattle, and large birds) (*13*). Consequently, the AHFV-positive ticks (except the 1 adult) most likely had fed only on the bird to which they were attached. This assumption implies that an immature tick could have acquired the virus vertically from its mother, from its putatively viremic avian host, or by co-feeding transmission, which can occur when ticks feed close to >1 infective tick (*14**,**15*). However, in this case, co-feeding transmission is less plausible because the 2 ticks attached to the same bird were not located close to each other (on the beak and nape). For several tickborne viruses, including KFDV, vertical transmission from the adult female tick to the eggs occurs. However, the prevalence of transovarial infection in nature is considered low (*14*). The 1 positive adult tick could have acquired virus in any of these ways or from a previous mammalian blood meal.

To our knowledge, birds have not been shown to play a role in the ecology or epidemiology of AHFV, and this tick species has not been identified previously as a possible vector. Our finding of this virus in ticks infesting birds en route from Africa to Europe and Asia, together with clinical cases in southern Egypt, the detection of AHFV RNA in ticks, and cases of seropositivity in Djibouti, could indicate a wider geographic distribution of the virus throughout eastern Africa and in novel regions. Further investigations of AHFV ecology and modes of transmission, including the potential role of *Hyalomma* ticks as vectors and passerine birds as reservoirs or distributors of potentially infected ticks, as well as increased surveillance, are needed.
